# Attitudes Toward Mathematics and Mathematics Teaching Anxiety: The Role of Mathematical Literacy, Self‐Efficacy, and Mathematical Thinking

**DOI:** 10.1002/brb3.71567

**Published:** 2026-06-25

**Authors:** Muhammed Celal Uras, Mehmet Şata, Yasin Soylu, Murat Yıldırım

**Affiliations:** ^1^ Department of Mathematics Education Agri Ibrahim Cecen University Ağrı Türkiye; ^2^ Department of Measurement and Evaluation Van Yüzüncü Yıl University Van Türkiye; ^3^ Department of Mathematics Education Atatürk University Erzurum Türkiye; ^4^ Department of Psychology Agri Ibrahim Cecen University Ağrı Türkiye; ^5^ Psychology Research Center Khazar University Baku Azerbaijan

**Keywords:** affective characteristics, attitudes toward mathematics, multiple mediation model, preservice mathematics teachers, teaching anxiety

## Abstract

**Objective:**

This study investigates the factors influencing preservice teachers’ anxiety about teaching mathematics within a theoretical framework. Drawing on Blömeke and Delaney's teacher competences model and Bandura's social cognitive theory, the research also incorporates expectancy‐value theory to examine the link between attitudes and anxiety. Specifically, the indirect effects of mathematical literacy self‐efficacy, mathematical thinking, and mathematical language self‐efficacy on mathematics teaching anxiety were explored through attitudes toward mathematics courses and mathematics education courses.

**Method:**

Data were collected from 689 preservice mathematics teachers (68.1% female). The proposed model was tested using four hypotheses through parallel mediation analysis.

**Results:**

Results indicated that mathematical literacy self‐efficacy, mathematical thinking, and mathematical language self‐efficacy exerted parallel mediating effects on the relationship between attitudes toward mathematics courses, attitudes toward mathematics education courses, and mathematics teaching anxiety. The findings further revealed that the inverse relationship between attitudes and anxiety was at least partially explained by these mediators.

**Conclusion:**

The study suggests the combined influence of self‐efficacy and attitudes in influencing preservice teachers’ anxiety levels, thereby enriching the theoretical framework of teacher competence. In light of Vygotsky's zone of proximal development, the findings also provide new insights into how the development of mathematical thinking and language skills can reduce teaching anxiety. Overall, the research highlights the importance of teacher education programs adopting a holistic approach that integrates both cognitive and affective dimensions.

## Graphical abstract

This study investigates how mathematical literacy self‐efficacy, mathematical thinking, and self‐efficacy of mathematical language indirectly influence mathematics teaching anxiety among pre‐service teachers, through their attitudes toward mathematics and mathematics education courses. A parallel mediation model tested on data from 689 pre‐service mathematics teachers confirmed that self‐efficacy and attitudes jointly shape anxiety levels. The findings support a multi‐theoretical framework (Teacher Competence Model, Social Cognitive Theory, Expectancy‐Value Theory, and Zone of Proximal Development) and highlight the need for teacher training programs to address both cognitive and emotional aspects of teaching mathematics.

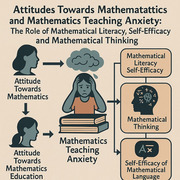

## 1 Introduction

Teacher training programs are important in training educators who are at the center of the education system. These programs aim to support preservice teachers (PSTs) in teaching skills by developing their professional competencies (Flores 2020). The spread of digitalization, diversification in student profiles, and increasing academic expectations require teachers to have strong competencies in pedagogical, affective, and cognitive domains (Leung et al. 2024). Mathematics teachers not only transfer mathematical knowledge. They also guide students' learning activities and influence their attitudes toward mathematics. In this regard, understanding teachers' professional competencies requires examining the anxiety, attitudes, and belief structures they encounter in their educational practices. The theoretical foundations of teachers' professional competencies have been explained through various models. Blömeke and Delaney's (2014) model of teacher competences provides an important framework. The model considers professional competence in two main dimensions: cognitive (content and pedagogical knowledge) and affective (motivational characteristics and beliefs). The interaction of these two dimensions shapes teachers' performances reflected in professional practices. In addition, Bandura's (2001) social cognitive theory and the concept of self‐efficacy beliefs reveal that individuals' perceptions of their achievements affect both their motivation to learn and their anxiety levels. Therefore, PSTs self‐efficacy beliefs, teaching anxieties, and mathematical thinking (MT) styles play a determining role in their perceptions of professional efficacy (Orakcı et al. 2023). Furthermore, expectancy‐value theory suggests that individuals' motivation toward a task depends on how valuable and important they personally find the task and their expectations of success in the task (Wigfield and Eccles 2000). It can be said that the constructs examined in the present study, such as attitude and anxiety, interact with PSTs self‐efficacy perceptions. The development of positive attitudes toward mathematics teaching depends on confidence in competence and low levels of anxiety (Cil and Akcay 2025). Vygotsky's proximal development theory argues that social interactions and language use are determinant in the process of realizing an individual's potential (Ness 2022). PSTs ability to use mathematical language is a key indicator of their ability to construct learning and guide students' learning. Mathematical language self‐efficacy is a social expression of an individual's cognitive development (Segura et al. 2025). In this respect, it can also be related to social cognitive theory.

In line with the theoretical foundations, the current study examines the mechanisms that play a role in the relationship between PSTs teaching anxiety and attitudes. However, few studies have examined the variables affecting these relationships or the structure they form (Karakose et al. 2023). In particular, the lack of models of the multiple interactions of different psychological and cognitive factors that may affect PSTs anxiety about teaching mathematics is remarkable. Current trends in theoretical frameworks indicate that variables such as mathematical literacy self‐efficacy (MLSE), MT, and self‐efficacy in mathematical language (SEML) may significantly explain the link between PSTs’ teaching anxieties and their attitudes. The present study provides a framework for understanding PSTs professional competences in the context of literacy, self‐efficacy, and ways of thinking by using multi‐mediated models. The focus of the study is to understand the cognitive and affective mechanisms behind mathematics teaching anxiety (MTA) and to develop strategic directions for teacher education. Therefore, the relationships between attitudes toward mathematics and teaching anxiety are examined through three main mediating variables: MLSE, MT, and self‐efficacy of mathematical language. It contributes both to the development of explanatory models for understanding PSTs professional competences and to the contemporary teacher education literature.

## 2 Literature Review

### 2.1 MTA

Anxiety describes personal feelings such as fear, panic, and worry (Craske et al. 2011). Furthermore, teaching anxiety refers to the fear experienced in teaching (Liu and Wu 2021). PSTs' anxiety about teaching mathematics, in addition to the lack of pedagogical knowledge, derives from their belief in mathematical competence and affective dispositions. Moreover, their anxiety about teaching mathematics is related to individual learning experiences, self‐efficacy perceptions, and attitudes toward mathematics (Papic and Papic 2025). In particular, low self‐efficacy and a negative attitude toward mathematics are important factors that increase teaching anxiety (Cil and Akcay 2025). Furthermore, the adoption of MT as a problem‐solving approach transforms the teaching process into a more structured and flexible form, which reduces the anxiety levels of PSTs. MLSE is also related to teaching anxiety. PSTs mathematical content knowledge also plays an important role in reducing anxiety (Aksu and Kul 2019). Therefore, these findings suggest that PSTs confidence in their mathematical knowledge and teaching skills significantly affects their teaching anxiety. Considering these variables together makes it necessary to evaluate teaching anxiety through affective, cognitive, and pedagogical dimensions.

### 2.2 Attitudes Toward Mathematics Courses (AMC)

Attitude refers to the emotional, cognitive, and behavioral tendencies of individuals toward a particular subject, and it is based on their evaluations and beliefs about the subject (Ajzen 2014). AMC includes emotional, cognitive, and behavioral tendencies toward mathematics. In addition to their mathematics achievement, PSTs attitudes toward mathematics courses are an important factor. These attitudes also influence their perceptions of competence in teaching mathematics (Chen et al. 2018). AMC can affect PSTs MTA (Perry et al. 2023). Furthermore, teaching anxiety increases when PSTs exhibit low self‐efficacy perceptions, poor MT skills, and negative attitudes (Novak and Tassell 2017). Attitudes toward mathematics have a significant effect on their MTA. AMC can significantly affect the quality of the teaching process. Therefore, these attitudes must be integrated into teacher training programs (Blömeke et al. 2011; König et al. 2021).

### 2.3 Attitude Toward Mathematics Education Courses (AMEC)

AMEC are important in the development of PSTs pedagogical content knowledge. These attitudes also have a role in the development of PSTs mathematical literacy, self‐efficacy, and MT skills (Yildiz et al. 2020). In particular, pedagogical content knowledge helps teachers to teach mathematical content effectively. It also helps them select the most appropriate teaching strategies for their students. Moreover, it plays a critical role in reducing PSTs anxiety toward the courses and improving their mathematical skills (Aksu and Kul 2019; Forsler et al. 2024). PSTs AMEC affect not only their mathematical knowledge but also their pedagogical skills (Hwang et al. 2018). In this context, considering factors such as mathematical literacy and self‐efficacy in teacher education programs plays an important role in the development of PSTs pedagogical content knowledge. Understanding the relationship between AMEC and teaching anxiety related to pedagogical content knowledge is important. It may require the integration of professional competences into teacher education programs.

### 2.4 Mathematics Literacy Self‐Efficacy

Mathematical literacy refers to the ability of individuals to understand mathematical concepts, explore the relationships between them, and use them effectively (Machaba 2017). Elements such as problem solving and interpretation skills form the basis of mathematical literacy (Thomson et al. 2013; Uras et al. 2024). Teachers’ mathematical literacy directly affects students’ achievement (Hwang and Ham 2021). Recent studies provide more data on how PSTs mathematical anxiety and literacy levels affect each other (Edo et al. 2024). However, the interrelationship of mathematical literacy with professional competences, in addition to attitudes and anxiety, has not been examined in depth. This deficiency may limit the effectiveness of interventions developed for PSTs education.

### 2.5 MT

MT refers to an individual's capacity to analyze, generalize, and make sense of mathematical situations. MT is among the variables that determine the quality of the teaching process (Güner and Erbay 2021). MT is necessary for problem solving or understanding concepts, and influences pedagogical decisions in teaching. Those with high self‐efficacy manage MT processes more effectively (Küçüktepe and Balkan 2021). Moreover, negative emotions (e.g., mathematics anxiety) can lead to a decline in both MT and mathematical literacy through self‐efficacy. This provides an understanding of how positive attitudes toward mathematics and strong self‐efficacy perceptions shape both PSTs MT skills and their teaching anxiety.

### 2.6 Self‐Efficacy of Mathematical Language

Mathematical language self‐efficacy is an individual's perception of personal competence in using mathematical concepts clearly, accurately, and coherently with appropriate symbols, terms, and expressions (Riccomini et al. 2015). Mathematical language self‐efficacy enables the transfer of concepts and the structuring of MT. Moreover, low mathematical language self‐efficacy can increase teaching anxiety, whereas high levels of efficacy can positively affect attitudes toward teaching (Bjerke and Xenofontos 2023). Therefore, mathematical language self‐efficacy is more than linguistic competence. It is a determining factor in reducing teaching anxiety and developing positive attitudes toward mathematics.

### 2.7 Exploring the Relationships Between Variables

To understand the complex structure of PSTs affective characteristics, it is important to recognize the intertwined relationships that exist between variables. The variables in the present study are components of a complex structure that influences a teacher's experience in the classroom. Teachers' emotional and cognitive reactions to teaching mathematics are referred to as teaching anxiety. MTA, central construct of the study, encompasses teachers' emotional and cognitive reactions to teaching mathematics (Brown et al. 2012). MTA can significantly affect the quality of classroom practice and students' achievement (Ramirez et al. 2018). Moreover, a teacher's attitudes toward both mathematics courses and pedagogical approaches play an important role in shaping teaching experiences. These attitudes are not only individual feelings but also interconnected. A positive attitude toward mathematics can lead to a better perception of the methods used to teach this subject (Hourigan et al. 2016). Positive attitudes contribute to a teacher's ability to teach more effectively and potentially alleviate teaching anxiety. Another factor that contributes to this is MLSE. A teacher's self‐confidence is based on his/her perception of MLSE. This belief in their ability to understand and communicate mathematical concepts is not only an independent characteristic. It is also a powerful force that interacts with other variables. A teacher with high MLSE is more likely to approach both mathematics content and pedagogy with confidence (Norton 2019). This, in turn, influences the teacher's attitude and acts as a guard against teaching anxiety. Despite all this, teachers may face several challenges and problems in teaching. To overcome these, MT skills are needed. MT encompasses the cognitive capacities to analyze and solve problems (Gao et al. 2022). A teacher's problem‐solving skills and logical reasoning are intertwined with their ability to explain concepts (Uras and Soylu 2024). This connection extends to their SEML, which affects how fluently they communicate complex ideas. As these skills support each other, they contribute to a teacher's overall competence in the classroom, affecting their confidence and therefore their level of teaching anxiety.

### 2.8 Present Study

This study is contextualized according to Blömeke and Delaney's teacher competences model, social cognitive theory, expectancy‐value theory, and Vygotsky's zone of convergent development theory. According to Blömeke and Delaney's teacher competences model, professional efficacy consists of cognitive (content and pedagogical knowledge) and affective (motivational characteristics and beliefs) dimensions. The interaction of these two dimensions influences teachers' performance in classroom practices. Social cognitive theory and the concept of self‐efficacy beliefs reveal that individuals' perceptions of success in various tasks affect both learning motivation and anxiety levels (Schunk and DiBenedetto 2020). Furthermore, expectancy‐value theory explains motivation based on the expectation of success (Nagle 2021). Attitude and anxiety interact with PSTs perceptions of self‐efficacy, which depends on developing positive attitudes toward mathematics teaching, efficacy confidence, and low anxiety levels. The zone of proximal development theory argues that social interactions and language use are decisive in the realization of an individual's potential. In this context, PSTs self‐efficacy to use mathematical language effectively is a key indicator of their ability to both construct their learning and guide others' learning. Beyond the theoretical framework, recent mathematics education research has focused on examining the competencies of teachers and PSTs and how they are transferred into classroom practice (Dervenis et al. 2022). The competence to teach mathematics determines the quality of mathematics teaching (Delgado‐Rebolledo and Zakaryan 2020). Moreover, there is a negative relationship between math anxiety and attitudes (Hunt and Maloney 2022). On the other hand, PSTs anxiety about teaching mathematics is a relatively new construct (Bosica 2022). In particular, the readiness of PSTs before they start their professional life should be examined according to different variables.

In the present study, variables that are thought to be directly or indirectly related to MTA were examined together. These variables are associated with both cognitive and affective dimensions of teacher competence. Therefore, it is also important to identify the variables that are effective in reaching a high level of competence of PSTs in their future professional life. The current study seeks to examine the mediating roles of MLSE, MT, and SEML in the relationship between affective professional characteristics. In this direction, a model has been proposed that includes the six constructs. To the best of our knowledge, there is no study in the literature that brings these six constructs together and examines their intertwined relationships in a single model. The present study is considered necessary because it examines these six constructs under a single model. In addition, it can be said that the present study is unique and essential because it identifies the constructs that mediate the relationship between MTA, AMC, and AMEC. Within this structural model, we proposed four main hypotheses:

**Hypothesis** **1**. *Attitude toward mathematics courses is related to mathematics teaching anxiety*.
**Hypothesis 2**. *Mathematical literacy self‐efficacy positively mediates the relationship between attitude toward mathematics courses and mathematics teaching anxiety*.
**Hypothesis 3**. *Mathematical thinking positively mediates the relationship between attitude toward mathematics courses and mathematics teaching anxiety*.
**Hypothesis 4**. *Self‐efficacy of mathematical language positively mediates the relationship between attitude toward mathematics courses and mathematics teaching anxiety*.

## 3 Method

### 3.1 Population Sample and Power Analysis

Power analysis was conducted to reveal the relationships accurately and robustly between the predictor and predicted variables. The conditions specified for power analysis were alpha level 0.05, beta level (second type error) 0.20, effect size low level, and two‐way hypothesis. The minimum sample size required under these conditions was identified as 485. Power analysis was performed through the GPower (version 3.1.9.7) package program. After the power analysis, 714 individuals were reached by the appropriate sampling method from the determined universe, and 25 individuals were excluded from the scope of the research due to missing data and outlier analysis. The study was conducted with 689 individuals. The power analysis was repeated for 689 individuals under the same conditions, and the test's power was 1 − *β* = 0.923. According to this result, it was determined that the analysis conducted on the sample had sufficient power. When examining the sociodemographic structure of the PSTs who voluntarily participated in the study, it was found that 68.1% were female, 31.9% were male, 27.9% were in the first grade, 35.8% were in the second grade, 18.6% in the third grade, and 17.7% were in the fourth grade. It was also found that the participants continued their education at 19 different universities in Turkey. The fact that the individuals participating in the study were selected from universities and grade levels in different regions of Turkey contributes to the generalizability of the study. Although the proportion of female participants in the sample was higher than the universal distribution, gender was not considered as an independent variable in the analyses. The model was tested on the whole sample.

### 3.2 Measures

#### 3.2.1 Mathematics Teaching Anxiety Scale (MATAS)

The 23‐item MATAS (Peker 2006) was developed to measure the MTA of PSTs. The items (e.g., “I feel as if I do not know anything about the mathematics topics I will teach”) are scored on a five‐point Likert scale from 1 (strongly disagree) to 5 (strongly agree). The score obtained from the scale varies between 23 and 115 points. An increase in the score obtained from the scale means an increase in PST's MTA, and a decrease in the score means a decrease in this anxiety. MATAS consists of four subscales. These are content knowledge, self‐confidence, attitude, and pedagogical knowledge. In the reliability and validity study conducted for this research, it was determined that the Cronbach *α* coefficient calculated for the subscales ranged between 0.834 and 0.926, and the stratified Cronbach *α* coefficient calculated for the whole scale was 0.951. A second‐order confirmatory factor analysis (CFA) was conducted based on the subdimensions of the MATAS scale. The model demonstrated an excellent fit to the data, as indicated by the following fit indices: RMSEA = 0.027, 90% CI (0.021, 0.033); SRMR = 0.048; GFI = 0.988; TLI = 0.994; NFI = 0.984; and CFI = 0.995.

#### 3.2.2 Attitude Scale for Courses in Mathematics (ASCIM)

The 20‐item ASCIM (Turanlı et al. 2008) was developed to measure PSTs AMC. The items (e.g., “Studying mathematics field courses makes me happy”) are scored on a five‐point Likert scale from 1 (strongly disagree) to 5 (strongly agree). The score obtained from the scale varies between 20 and 100 points. An increase in the score obtained from the scale means an increase in PST's AMC, and a decrease in the score means a decrease in these attitudes. In the reliability and validity study conducted for this research, the Cronbach *α* coefficient calculated for the whole scale was 0.923. A CFA was performed for the ASCIM scale. The model yielded acceptable to good fit indices: RMSEA = 0.062, 90% CI (0.057, 0.068); SRMR = 0.080; GFI = 0.971; TLI = 0.964; NFI = 0.956; and CFI = 0.968.

#### 3.2.3 Attitude Scale for Mathematics Education Courses (ASMEC)

The 20‐item ASMEC (Karakaş‐Türker and Turanlı 2008) was developed to measure PSTs AMEC. The items (e.g., “I need to be successful in mathematics education courses”) are scored on a five‐point Likert scale from 1 (strongly disagree) to 5 (strongly agree). The score obtained from the scale varies between 18 and 90 points. An increase in the score obtained from the scale means an increase in PST's AMEC, and a decrease in the score means a decrease in these attitudes. In the reliability and validity study conducted for this research, the Cronbach *α* coefficient calculated for the whole scale was 0.932. A CFA was conducted for the ASMEC scale. The model demonstrated a good fit to the data, as evidenced by the following fit indices: RMSEA = 0.046, 90% CI (0.039, 0.052); SRMR = 0.065; GFI = 0.984; TLI = 0.984; NFI = 0.977; and CFI = 0.986.

#### 3.2.4 Self‐Efficacy Scale of Mathematics Literacy (SSML)

The 25‐item SSML (Özgen and Bindak 2008) was developed to measure PST's MLSE. The items (e.g., “I can see mathematical relationships in scientific phenomena”) are scored on a five‐point Likert scale from 1 (strongly disagree) to 5 (strongly agree). The score obtained from the scale varies between 25 and 125 points. An increase in the score obtained from the scale means an increase in PST's self‐efficacy perceptions about mathematical literacy, and a decrease in the score means a decrease in this self‐efficacy perception. In the reliability and validity study conducted for this research, the Cronbach *α* coefficient calculated for the whole scale was 0.942. A CFA was conducted for the SSML scale. The model demonstrated a good fit to the data, as evidenced by the following fit indices: RMSEA = 0.041, 90% CI (0.036, 0.045); SRMR = 0.059; GFI = 0.986; TLI = 0.989; NFI = 0.982; and CFI = 0.990.

#### 3.2.5 MT Scale (MTS)

The 25‐item MTS (Ersoy and Başer 2013) was developed to measure PST's MT levels. The items (e.g., “Individuals with creative thinking skills acquire mathematical thinking skills more easily”) are scored on a five‐point Likert scale from 1 (strongly disagree) to 5 (strongly agree). The score obtained from the scale varies between 25 and 125 points. An increase in the score obtained from the scale means an increase in the MT level of PST, and a decrease in the score means a decrease in the MT level. MTS consists of four subscales. These are high‐level tendencies to thinking, reasoning, problem‐solving, and MT. In the reliability and validity study conducted for this research, it was determined that the Cronbach *α* coefficient calculated for the subscales ranged between 0.573 and 0.838 and the stratified Cronbach *α* coefficient calculated for the whole scale was 0.887. A second‐order CFA was conducted to evaluate the factorial structure of the MTS scale based on its subdimensions. The model showed a good fit to the data, as indicated by the following fit indices: RMSEA = 0.056, 90% CI (0.051, 0.060); SRMR = 0.067; GFI = 0.971; TLI = 0.967; NFI = 0.957; and CFI = 0.971.

#### 3.2.6 Teacher Self‐Efficacy Scale in Language of Mathematics (TSESLoM)

The 17‐item TSESLoM (Kabael and Yayan 2017) was developed to measure PST's SEML levels toward using and teaching mathematical language. The items (e.g., “I can use mathematical language to express mathematical ideas”) are scored on a four‐point Likert scale from 1 (strongly disagree) to 4 (strongly agree). The score obtained from the scale varies between 17 and 68 points. An increase in the score obtained from the scale means an increase in PST's self‐efficacy perception level toward using and teaching mathematical language. In contrast, a decrease in the score means a decrease in this self‐efficacy perception level. TSESLoM consists of three subscales. These are teaching mathematical language, specific use of mathematical language, and general use of mathematical language. In the reliability and validity study conducted for this research, it was determined that the Cronbach *α* coefficient calculated for the subscales ranged between 0.715 and 0.813 and the stratified Cronbach *α* coefficient calculated for the whole scale was 0.884. A second‐order CFA was performed to examine the factorial structure of the TSESLoM scale based on its underlying subdimensions. The model demonstrated an acceptable fit to the data, as reflected in the following fit indices: RMSEA = 0.081, 90% CI (0.074, 0.088); SRMR = 0.085; GFI = 0.964; TLI = 0.935; NFI = 0.935; and CFI = 0.946.

### 3.3 Procedure

The present study employed a quantitative research design, and data were collected using an online survey method. The questionnaire was created using Google Forms. Participants were selected using convenience sampling. All participants were informed about the purpose and procedures of the study. They were also asked for confirmation before completing the questionnaires. An online link to the survey was sent to the participants, and each participant was allowed to complete the survey only once. Moreover, three items instructed participants (e.g., “Please mark Option 1 in this question”) to determine screening question errors. Confidentiality and anonymity of responses were assured for all participants. Participation in the study was entirely voluntary. Ethics committee approval of this research was obtained from Ağrı İbrahim Çeçen University (reference number: 167), and every research stage was carried out in accordance with the Declaration of Helsinki.

### 3.4 Data Analysis

According to the purpose of the study and the nature of the data, descriptive statistics were first used in data analysis. Then, Cronbach's alpha and stratified Cronbach's alpha coefficients were used to provide evidence of the reliability of the measurements obtained from the measurement tools. At the same time, CFA was performed to provide evidence of the validity of the measurements obtained from the measurement tools. Skewness and kurtosis values were reported to determine the distribution of the measurements obtained from the measurement tools. Finally, regression‐based mediation analysis was carried out for the mediation model, which is the purpose of the study. In the study, the value of 0.05 was used for statistical significance.

## 4 Findings

### 4.1 Descriptive Statistics and Correlation Analysis

Descriptive statistics (e.g., mean, standard deviation, normality tests) of the measurements for the variables are presented in Table 1. The skewness and kurtosis values of the measurement tools used in the research are within the range of ±1.000. Accordingly, it was determined that the distributions of the measurement tools were found to be close to the normal distribution (Shiel and Cartwright 2015). It is seen that the averages of the attitude scale for mathematics courses, the attitude scale for mathematics education courses, the SSML, the MTS, and the TSESLoM are high, and the average of the MATAS is low.

**TABLE 1:** Correlations between variables and descriptive statistics.

Variables	Descriptive statistics				Correlations					
	Mean	SD	Skew.	Kurt.	1	2	3	4	5	6
1. Attitude toward mathematics courses	76.42	13.68	−0.66	0.48	—					
2. Attitude toward mathematics education courses	71.33	12.51	−0.73	0.60	0.50**	—				
3. Mathematical literacy self‐efficacy	92.41	15.12	−0.17	−0.27	0.39**	0.47**	—			
4. Mathematical thinking	97.49	11.26	−0.36	−0.56	0.37**	0.52**	0.57**	—		
5. Self‐efficacy of mathematical language	52.35	7.83	0.23	−0.73	0.41**	0.44**	0.65**	0.52**	—	
6. Mathematics teaching anxiety	45.01	13.85	0.42	−0.61	−0.51**	−0.59**	−0.65**	−0.58**	−0.68**	—

Abbreviations: Kurt., kurtosis; SD, standard deviation; Skew., skewness.

*p ˂ 0.05; **p ˂ 0.001.

The Pearson correlation coefficient was calculated to determine the relationship between the measurement tools. The results of the correlation analysis showed that all the variables in the study have statistically significant relationships with each other (*p* ˂ 0.05). MATAS, which was considered a predicted variable in the study, was found to have a negative and moderate relationship with the predictor (ASCIM and ASMEC) and mediator (SSML, MTS, TSESLoM) variables. The lowest relationship is between the MTS and the attitude scale for mathematics courses, while the highest is between the TSESLoM and the MATAS. All variables show statistically significant relationships with each other, which is sufficient for establishing the mediation model.

### 4.2 Mediation Analysis

A regression‐based mediation analysis was conducted, and the findings are presented in Table 2.

**TABLE 2:** Indirect, direct, and total effects of mediation analysis.

Path	Effect	Estimate	95% CI		*β*	*p*
			Lower	Upper		
Indirect	ASCIM ⇒ SSML ⇒ MATAS	−0.05	−0.07	−0.03	−0.05	< 0.001
	ASCIM ⇒ MTS ⇒ MATAS	−0.02	−0.03	−0.01	−0.02	0.002
	ASCIM ⇒ TSESLoM ⇒ MATAS	−0.08	−0.11	−0.05	−0.08	< 0.001
	ASMEC ⇒ SSML ⇒ MATAS	−0.09	−0.12	−0.06	−0.08	< 0.001
	ASMEC ⇒ MTS ⇒ MATAS	−0.07	−0.10	−0.04	−0.06	< 0.001
	ASMEC ⇒ TSESLoM ⇒ MATAS	−0.11	−0.14	−0.08	−0.11	< 0.001
Component	ASCIM ⇒ SSML	0.23	0.14	0.31	0.21	< 0.001
	SSML ⇒ MATAS	−0.20	−0.25	−0.15	−0.23	< 0.001
	ASCIM ⇒ MTS	0.12	0.06	0.18	0.14	< 0.001
	MTS ⇒ MATAS	−0.17	−0.23	−0.10	−0.14	< 0.001
	ASCIM ⇒ TSESLoM	0.14	0.10	0.19	0.25	< 0.001
	TSESLoM ⇒ MATAS	−0.56	−0.65	−0.47	−0.33	< 0.001
	ASMEC ⇒ SSML	0.44	0.35	0.53	0.36	< 0.001
	ASMEC ⇒ MTS	0.40	0.34	0.47	0.45	< 0.001
	ASMEC ⇒ TSESLoM	0.20	0.15	0.25	0.32	< 0.001
Direct	ASCIM ⇒ MATAS	−0.15	−0.20	−0.09	−0.15	< 0.001
	ASMEC ⇒ MATAS	−0.22	−0.29	−0.15	−0.21	< 0.001
Total	ASCIM ⇒ MATAS	−0.29	−0.36	−0.23	−0.29	< 0.001
	ASMEC ⇒ MATAS	−0.49	−0.56	−0.42	−0.44	< 0.001

Abbreviations: ASCIM, attitude toward mathematics courses; ASMEC, attitude toward mathematics education courses; MATAS, mathematics teaching anxiety; MTS, mathematical thinking; SSML, self‐efficacy of mathematical language; TSESLoM, teacher self‐efficacy scale in language of mathematics.

Table 2 shows that there was a statistically significant relationship between ASCIM and MATAS scores (total effect = −0.29, 95% CI = [−0.36, −0.23], *p* < 0.001). Similarly, PSTs ASMEC and MATAS scores were found to have a statistically significant relationship (total effect = −0.49, 95% CI = [−0.56, −0.42], *p* < 0.001). ASCIM is also a positive predictor of SSML (effect = 0.23, 95% CI = [0.14, 0.31], *p* < 0.001), MTS (effect = 0.12, 95% CI = [0.06, 0.18], *p* < 0.001), and TSESLoM (effect = 0.14, 95% CI = [0.10, 0.19], *p* < 0.001). Similarly, ASMEC is also a positive predictor of SSML (effect = 0.44, 95% CI = [0.35, 0.53], *p* < 0.001), MTS (effect = 0.40, 95% CI = [0.34, 0.47], *p* < 0.001), and TSESLoM (effect = 0.20, 95% CI = [0.15, 0.25], *p* < 0.001). In addition, SSML (effect = −0.20, 95% CI = [−0.25, −0.15], *p* < 0.001), MTS (effect = −0.17, 95% CI = [−0.23, −0.10], *p* < 0.001), and TSESLoM (effect = −0.56, 95% CI = [−0.65, −0.47], *p* < 0.001) were negative predictors of MATAS.

When indirect effects were examined, there was a significant indirect effect of ASCIM on MATAS via SSML (indirect effect = −0.05, 95% CI = [−0.07, −0.03], *p* < 0.001). Also, the indirect effect of ASCIM on MATAS via MTS was significant (indirect effect = −0.02, 95% CI = [−0.03, −0.01], *p* < 0.05). Lastly, the indirect effect of ASCIM on MATAS via TSESLoM was significant (indirect effect = −0.08, 95% CI = [−0.11, −0.05], *p* < 0.001). Moreover, indirect effects were examined; there was a significant indirect effect of ASMES on MATAS via SSML (indirect effect = −0.09, 95% CI = [−0.12, −0.06], *p* < 0.001). Also, the indirect effects of ASMEC on MATAS via MTS were tested. The effect was significant (indirect effect = −0.07, 95% CI = [−0.10, −0.04], *p* < 0.05). Moreover, the indirect effects of ASMEC on MATAS via TSESLoM were tested. The effect was significant (indirect effect = −0.11, 95% CI = [−0.14, −0.08], *p* < 0.001). In the relationship between ASCIM and MATAS, SSML, MTS, and TSESLoM had parallel mediating effects. Furthermore, in the relationship between ASMEC and MATAS, SSML, MTS, and TSESLoM had parallel mediating effects.

**FIGURE 1:**
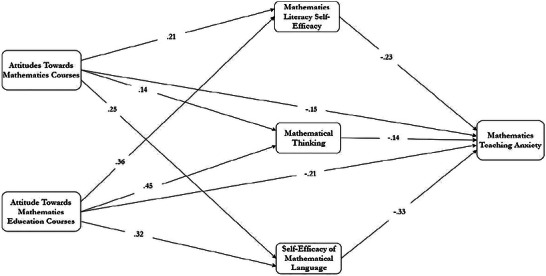
Illustration of the mediation model. *Note*: All effects were significant. Indirect and total effects are not shown in the figure.

The results indicated that ASCIM and ASMEC predicted MATAS. When ASCIM was entered as the predictor, it significantly predicted MATAS (*β* = −0.29, *p* < 0.001) and accounted for 1.46% of the variance in the model. When ASMEC was entered as the predictor, it significantly predicted MATAS (*β* = −0.44, *p* < 0.001) and accounted for 2.4% of the variance in the model. In addition, results indicated that SSML, MTS, and TSESLoM predicted MATAS. When SSML was entered as the predictor, it significantly predicted MATAS (*β* = −0.23, *p* < 0.001) and accounted for 2.37% of the variance in the model. When MTS was entered as the predictor, it significantly predicted MATAS (*β* = −0.14, *p* < 0.001) and accounted for 1.08% of the variance in the model. When TSESLoM was entered as the predictor, it significantly predicted MATAS (*β* = −0.33, *p* < 0.001), and accounted for 5.29% of the variance in the model. Among the indirect paths analyzed, the effect of the ASMEC variable on MATAS through TSESLoM stands out as the strongest and most significant path. The indirect effect obtained through this pathway was statistically significant (*β* = −0.11, 95% CI [−0.14, −0.08]) and reflects a remarkable negative relationship. The figural representation of the tested mediation model is shown in Figure 1.

The relationships between the dependent and independent variables are all statistically significant (see Figure 1). For simplicity, indirect and total effects are not included in the model and are presented in Table 2.

## 5 Discussion

The present study examined the mediating role of MLSE, MT, and SEML in the relationship between AMC, AMEC, and MTA. The findings also support the theoretical perspectives that explain teacher efficacy through the interaction of cognitive and affective dimensions. In this context, the study was discussed within the framework of Blömeke and Delaney's teacher competences model, Bandura's social cognitive theory, Eccles and Wigfield's expectancy‐value theory, and Vygotsky's zone of proximal development theory. The results are interpreted in terms of both theoretical and empirical support. Consistent with Hypothesis 1, findings showed that AMC predicted MTA. In other words, AMC had a significant negative effect on MTA. Considering that anxiety is related to reduced attitudes, it can be said that the finding aligns with the literature (Papic and Papic 2025). Similarly, AMEC predicted MTA. It is consistent with the findings of the studies that reveal the relationship between teaching anxiety and pedagogical dimension (Aksu and Kul 2019). According to the present study's findings, it was found that MLSE and MT, as well as SEML, predicted MTA. This finding is consistent with the literature (Bjerke and Xenofontos 2023; Edo et al. 2024; Rabab'h 2015). PSTs need to improve their literacy, language self‐efficacy, and thinking skills to manage their MTA. Those with high levels of teaching anxiety may face challenges in effectively teaching mathematics (Patkin and Greenstein 2020; Peker and Ulu 2018). According to these findings, it can be said that a decrease in MLSE, MT, and SEML would increase MTA. Therefore, the results of this study are consistent with this relationship.

The results show that as hypothesized, MLSE (Hypothesis 2) and MT (Hypothesis 3), and SEML (Hypothesis 4) partially mediate the relationship between AMC, AMEC, and MTA. In other words, AMC and AMEC increase MLSE, MT, and SEML, which decreases PSTs MTA. This result supports the fundamental principles of Blömeke and Delaney's model, conceptualizing teacher efficacy as a multidimensional construct. According to this model, teacher efficacy is a multidimensional construct shaped by the interaction of cognitive, affective, and motivational components. The finding that self‐efficacy perception was the strongest predictor of teaching anxiety supports this holistic approach. First, AMC and AMEC directly and indirectly predict PSTs MTA. Positive attitudes toward mathematics and mathematics education courses enhance their confidence in their mathematical abilities. This confidence helps them handle teaching challenges with lower levels of anxiety. This finding supports the self‐efficacy belief–anxiety relationship, which is one of the basic principles of social cognitive theory (Bandura 2001). Empirical research has also shown that positive attitudes reduce teaching anxiety through self‐efficacy perception (Orozco‐Guzmán et al. 2020). As a result of the findings, Hypothesis 2 was confirmed; both AMC and AMEC positively predicted MLSE and significantly reduced MTA. This finding is consistent with the determinant effect of the cognitive efficacy component emphasized in Blömeke and Delaney's model on teacher behaviors (Blömeke and Delaney 2014). Individuals who can construct mathematical content in a meaningful way can confidently transfer knowledge in the classroom environment. This contributes to the reduction of instructional anxiety (Rabab'h 2015). Similarly, MT has a mediating role in the relationship between AMC, AMEC, and MTA (Hypothesis 3 was confirmed). PSTs' thinking skills may influence their teaching anxiety, especially given the role that thinking styles such as problem solving, reasoning, and generalization play in teaching processes. Consistent with the results of Ahmat et al. (2022), PSTs with high MT competence are better able to manage uncertainties related to the teaching process, which reduces their anxiety levels. As Hypothesis 4 was confirmed, the most remarkable finding was that SEML emerged as the strongest mediating variable in the relationship between both AMC and AMEC and MTA. This finding is related to Vygotsky's theoretical approach that learning is structured through language (Rigopouli et al. 2025). Proficiency in mathematical language enables PSTs to convey conceptual content accurately and participate confidently in student interactions. Novak and Tassell (2017) emphasize that individuals with low mathematical language proficiency have more communication problems and related anxiety during teaching. Therefore, the emergence of SEML as a mediator in this study points to the necessity of systematic interventions for linguistic competencies in teacher education programs.

### 5.1 Implications

Findings emphasize that cognitive‐affective components such as MLSE, MT, and SEML stand out as mediating variables in the relationship between PSTs' attitudes toward mathematics and mathematics education courses and anxiety about teaching mathematics. This result provides a strong example of the influence of an individual's beliefs on affective reactions and behaviors within the framework of social cognitive theory (2001). The negative relationship between self‐efficacy beliefs and teaching anxiety was confirmed by the mediation structure modeled in this study. It implies that teacher education programs must incorporate multidimensional structures to strengthen the cognitive (e.g., MT), linguistic (e.g., mathematical language proficiency), and self‐efficacy dimensions of PSTs, moving beyond focus on knowledge acquisition. These programs can include mathematical language modules to reinforce the transfer of concepts and terms and promote the use of descriptive language. The programs may also include thinking‐promoting practices aimed at developing reasoning, generalization, and logical skills through problem‐based activities. They can incorporate additional practices to support literacy skills by applying mathematical content to daily life contexts. Integrating these components with micro‐teaching simulations and feedback sessions enhances both cognitive and affective competencies, ultimately reducing PSTs’ MTA.

### 5.2 Limitations and Directions for Further Studies

This study has several limitations. First, the data collected in this study were collected using different measurement tools based on the participants' statements. This means that the data collected is limited by the scope of the measurement instruments. In addition, the model that emerged from the analysis shows that AMC and AMEC predict MLSE, MT, and SEML. Therefore, longitudinal and experimental studies are needed to analyze this causal relationship in more detail. Researchers conduct studies aimed at increasing PSTs’ AMC and AMEC. To support this development, teacher education programs should incorporate targeted activities that foster positive attitudes in these areas. Second, the gender distribution within the sample was unbalanced, with a higher representation of females (68.1%) compared to males (31.9%). This skewed distribution could potentially introduce gender‐related biases and limit the generalizability of the results to a wider population. Third, the reliance on self‐reported measures is another limitation. Self‐report instruments provide valuable information about an individual's subjective experiences. However, they are susceptible to response biases, recall inaccuracies, and social desirability effects.

## 6 Conclusion

This study found that AMC and AMEC are predictors of MTA based on MLSE, MT, and SEML. Positive attitudes toward mathematics and mathematics education courses strongly enhance PSTs’ MLSE, MT, and SEML. They also play an important role in reducing PSTs’ MTA. The order of importance of the mediating variables was found to be self‐efficacy of mathematical language, mathematics literacy self‐efficacy, and MT. In conclusion, this study suggests that teacher education programs require more comprehensive, cognitive‐affective–based, and structured intervention strategies. Further studies, supported by longitudinal and experimental methods, will enable the model presented here to be tested more comprehensively and contribute to practical policy development processes.

## Author Contributions

**Muhammed Celal Uras**: conceptualization, investigation, writing – original draft, visualization. **Mehmet Şata**: conceptualization, methodology, investigation, data curation, writing – original draft, visualization, validation. **Yasin Soylu**: conceptualization, writing – review and editing, supervision. **Murat Yıldırım**: writing – review and editing, supervision.

## Funding

The authors have nothing to report.

## Ethics Statement

All procedures performed in studies involving human participants were in accordance with the ethical standards of the institutional and/or national research committee and with the 1964 Helsinki Declaration and its later amendments or comparable ethical standards.

## Consent

Consent was obtained from all participants included in the study.

## Conflicts of Interest

The authors declare no conflicts of interest.

## Data Availability

The datasets generated and/or analyzed during the current study are available from the corresponding author upon reasonable request.
